# Supplementary material for: Examining the Association Between Overweight, Obesity, and Irritable Bowel Syndrome: A Systematic Review and Meta-Analysis
Source: Nutrients. 2024 Nov 21;16(23):3984. doi: 10.3390/nu16233984 (PMC11643418; doi:10.3390/nu16233984)
Supplement: Supplementary file 1 [file nutrients-16-03984-s001.zip › 20241112 ibs obesity supp.pdf]

Supplementary Material

Table S1: Full search strategy for the various databases

**Database**	**Search Strategy**	
Medline	Obesity/overweight	exp Obesity/ or exp Overweight/ or exp Body Mass Index/ or (obesity or obese* or overweight or over-weight or BMI or "body mass index").ti,ab.
	IBS	exp Irritable Bowel Syndrome/ or ("Irritable Bowel" or IBS or "Irritable Colon" or "Mucous Colitis" or "Spastic Colitis" or "Spastic Colon").ti,ab.
	Filter	English language, 1989 till current
Embase	Obesity/overweight	‘obesity'/exp OR 'body mass'/exp OR obesity:ti,ab OR obese*:ti,ab OR overweight:ti,ab OR 'over weight':ti,ab OR bmi:ti,ab OR 'body mass index':ti,ab
	IBS	'irritable colon'/exp OR 'irritable bowel':ti,ab OR ibs:ti,ab OR 'irritable colon':ti,ab OR 'mucous colitis':ti,ab OR 'spastic colitis':ti,ab OR 'spastic colon':ti,ab
	Article filter	'article'/it AND [1989-2024]/py AND [english]/lim
Cochrane Library	#1	MeSH descriptor: [Irritable Bowel Syndrome] explode all trees
	#2	((“Irritable Bowel” or IBS or “Irritable Colon” or “Mucous Colitis” or “Spastic Colitis”)):ti,ab,kw
	#3	#1 or #2
	#4	MeSH descriptor: [Obesity] explode all trees

	#5	MeSH descriptor: [Overweight] explode all trees
	#6	MeSH descriptor: [Body Mass Index] explode all trees
	#7	((obesity or obese* or overweight or over-weight or BMI or "body mass index")):ti,ab,kw
	#8	#4 or #5 or #6 or #7
	#9	#3 and #8

Table S2: Detailed breakdown of the risk of bias assessment for studies reviewed

Cross-sectional studies assessed using the Agency for Healthcare Research and Quality checklist

Criteria/Study	1. Clearly stated objective?	2. Defined study population?	3. Inclusion/exclusion criteria specified?	4. Time period defined?	5. Validity/reliability of measures mentioned?	6. Study design accounted for confounding?	7. Detailed methods for data collection?	8. Outcome measures clearly defined?	9. Statistical methods described?	10. Reporting of results (point estimates & variability)?	11. Limitations of study discussed?	Total Score
Anthea	1	1	0	1	1	0	1	1	0	0	1	7
Aljammaz	1	1	1	1	1	0	1	1	1	0	0	8
Alqahtani	1	1	1	1	1	0	1	1	1	0	0	8
Thomas-Dupont	1	1	1	1	1	0	1	1	1	0	0	8
Das	1	1	1	1	1	0	1	1	1	1	0	9
Akhondi	1	1	0	0	1	1	1	1	1	1	1	9

Alvand	1	1	1	1	1	0	1	1	1	0	1	9
Buscail	1	1	1	1	1	0	1	1	1	0	1	9
Khalil	1	1	1	1	1	0	1	1	1	0	1	9
Siah	1	1	0	0	1	1	1	1	1	1	1	9
Jadallah	1	1	1	1	1	0	1	1	1	1	1	10
Junkka	1	1	1	0	1	1	1	1	1	1	1	10
Mohammed	1	1	1	1	1	1	1	1	1	0	1	10
Alharbi, 2022	1	1	1	1	1	1	1	1	1	1	1	11
Bouchoucha, 2016	1	1	1	1	1	1	1	1	1	1	1	11
Fujiwara	1	1	1	1	1	1	1	1	1	1	1	11

Le Pluart	1	1	1	1	1	1	1	1	1	1	1	11
Roth	1	1	1	1	1	1	1	1	1	1	1	11
Camacho	1	1	1	1	1	1	1	1	1	1	1	11
Nilsson	1	1	1	1	1	1	1	1	1	1	1	11
Schmulson	1	1	0	0	0	0	1	1	1	0	1	6

Case-control and cohort studies assessed with the Newcastle-Ottawa Scale

Criteria/Study	Is the case definition adequate?	Representativeness of the cases	Selection of Controls	Definition of Controls	Comparability of cases and controls on the basis of the design or analysis	Ascertainment of exposure	Same method of ascertainment for cases and controls	Non-Response rate	Total score
Bayrak	1	1	0	1	2	0	1	1	7
Brochard	1	1	1	1	2	0	1	1	8
Farzaneh	1	1	0	1	1	1	1	1	7
Guo	1	1	1	1	1	1	1	1	8

Melchior	1	1	1	1	2	1	1	1	9
Carter	1	1	1	1	2	1	1	1	9

Table S3: List of articles deemed ineligible in the full text review

Authors	Year	Title	Reasons for exclusion
M. Aasbrenn; J. Valeur; P. G. Farup	2018	Evaluation of a faecal dysbiosis test for irritable bowel syndrome in subjects with and without obesity	No usable data
M. I. Abdalla; R. S. Sandler; M. D. Kappelman; C. F. Martin; W. Chen; K. Anton; M. D. Long	2017	Prevalence and Impact of Inflammatory Bowel Disease-Irritable Bowel Syndrome on Patient-reported Outcomes in CCFA Partners	No usable data
V. Ahuja; A. Agarwal; S. Gupta; P. Kumar	2021	Pattern of Irritable Bowel Syndrome and its Symptoms Characteristics: A Study from Tertiary Care Hospital in North India	No usable data
A. E. Almazar; J. Y. Chang; J. J. Larson; E. J. Atkinson; G. R. Locke; N. J. Talley; Y. A. Saito	2019	Comparison of Lactase Variant MCM6-13910 C>T Testing and Self-report of Dairy Sensitivity in Patients with Irritable Bowel Syndrome	No usable data
T. Bai; J. Xia; Y. Jiang; H. Cao; Y. Zhao; L. Zhang; H. Wang; J. Song; X. Hou	2017	Comparison of the Rome IV and Rome III criteria for IBS diagnosis: A cross-sectional survey	No usable data
A. Berstad; R. Undseth; R. Lind; J. Valeur	2012	Functional bowel symptoms, fibromyalgia and fatigue: A food-induced triad?	No usable data
I. K. Blom-Høgestøl; M. Aasbrenn; M. Chahal-Kummen; C. Brunborg; I. Eribe; J. Kristinsson; P. G. Farup; T. Mala	2019	Irritable bowel syndrome-like symptoms and health related quality of life two years after Roux-en-Y gastric bypass - A prospective cohort study	No usable data
I. K. Blom-Høgestøl; A. Stubhaug; J. A. Kristinsson; T. Mala	2018	Diagnosis and treatment of chronic abdominal pain 5 years after Roux-en-Y gastric bypass	No usable data
R. K. Breckan; A. M. Asfeldt; B. Straume; J. Florholmen; E. J. Paulssen	2012	Prevalence, comorbidity, and risk factors for functional bowel symptoms: a population-based survey in Northern Norway	No usable data
D. M. Brenner; S. D. Dorn; R. P. Fogel; J. Christie; A. P. Laitman; J. Rosenberg	2023	Plecanatide Improves Symptoms of Irritable Bowel Syndrome with Constipation: Results of an Integrated Efficacy and Safety Analysis of Two Phase 3 Trials	No usable data
D. M. Brenner; A. Sharma; S. S. C. Rao; A. P. Laitman; Z. Heimanson; C. Allen; G. S. Sayuk	2024	Plecanatide Improves Abdominal Bloating and Bowel Symptoms of Irritable Bowel Syndrome with Constipation	No usable data
M. Camilleri; M. Vazquez-Roque; J. Iturrino; A. Boldingh; D. Burton; S. McKinzie; B. S. Wong; A. S. Rao; E. Kenny; M. Månsson; et al.	2012	Effect of a glucagon-like peptide 1 analog, ROSE-010, on GI motor functions in female patients with constipation-predominant irritable bowel syndrome	No usable data
D. Carter; M. Beer-Gabel; E. Derazne; D. Tzur; E. Bardan	2017	The Severity of Symptoms Related to Irritable Bowel Syndrome is a Risk Factor for the Misclassification of Significant Organic Disease	No usable data

H. C. Chang; A. M. F. Yen; J. C. Y. Fann; S. Y. H. Chiu; C. S. Liao; H. H. Chen; K. C. Yang; L. S. Chen; Y. M. Lin	2015	Irritable bowel syndrome and the incidence of colorectal neoplasia: A prospective cohort study with community-based screened population in Taiwan	No usable data
K. Cheng; C. Lee; R. Garniene; H. Cabral; H. C. Weber	2024	Epidemiology of Irritable Bowel Syndrome in a Large Academic Safety-Net Hospital	No usable data
I. Cifci; O. Gokdemir; O. Aygun; D. Guldal	2023	Evaluation of functional constipation frequency and related factors	No usable data
M. Daher; S. Abbas; Z. Asaad; K. Khalil; G. Jadid	2024	Prevalence of fibromyalgia and irritable bowel syndrome and its association with studying medicine, a cross-sectional study in Al-Baath University, Syria	No usable data
J. J. De Jong; C. S. S. Latenstein; D. Boerma; E. J. Hazebroek; D. Hirsch; J. T. Heikens; J. Konsten; F. Polat; M. A. Lantinga; C. J. H. M. Van Laarhoven; J. P. H. Drenth; P. R. De Reuver	2022	Functional Dyspepsia and Irritable Bowel Syndrome are Highly Prevalent in Patients with Gallstones and Are Negatively Associated with Outcomes after Cholecystectomy: A Prospective, Multicenter, Observational Study (PERFECT-Trial)	No usable data
A. P. M. de MendonÇA; L. M. Yamashita; E. D. Silva; I. Solar; L. A. O. Santos; A. C. J. Vasques	2020	Nutritional status, quality of life and life habits of women with irritable bowel syndrome: A case-control study	No usable data
H. Dejong; S. Perkins; M. Grover; U. Schmidt	2011	The prevalence of irritable bowel syndrome in outpatients with bulimia nervosa	No usable data
Y. Dogan Kaya; A. Uzuner	2022	The relationship between abdominal obesity and irritable bowel syndrome in adults	No usable data
Y. Dong; S. Berens; W. Eich; R. Schaefert; J. Tesarz	2018	Is body mass index associated with symptom severity and health-related quality of life in irritable bowel syndrome? A cross-sectional study	No usable data
A. S. Erdinc; E. K. Aksoy; F. P. Sapmaz; D. Dikmen	2021	The dietary fibre intake of patients with constipation-predominant irritable bowel syndrome who applied different medical nutrition therapies	No usable data
E. Eslampour; K. Ghanadi; V. Aghamohammadi; A. M. Kazemi; R. Mohammadi; F. Vahid; A. Abbasnezhad	2021	"Association between dietary inflammatory index (DII) and risk of irritable bowel syndrome: a case-control study"	No usable data
A. C. Ford; D. Forman; A. G. Bailey; A. T. Axon; P. Moayyedi	2007	Initial poor quality of life and new onset of dyspepsia: results from a longitudinal 10-year follow-up study	No usable data
G. S. Fosnes; S. Lydersen; P. G. Farup	2011	Constipation and diarrhoea - common adverse drug reactions? A cross sectional study in the general population	No usable data

A. N. Galica; R. Galica; D. L. Dumitrascu	2021	Epidemiology of irritable bowel syndrome in albania	No usable data
S. Gallas; H. Knaz; J. Methnani; M. Maatallah Kanzali; A. Koukane; M. H. Bedoui; I. Latiri	2022	Prevalence and risk factors of functional gastrointestinal disorders in early period medical students: a pilot study in Tunisia	No usable data
X. Gao; S. Tian; N. Huang; G. Sun; T. Huang	2024	Associations of daily sedentary behavior, physical activity, and sleep with irritable bowel syndrome: A prospective analysis of 362,193 participants	No usable data
Y. Guo; K. Niu; H. Momma; Y. Kobayashi; M. Chujo; A. Otomo; S. Fukudo; R. Nagatomi	2014	Irritable bowel syndrome is positively related to metabolic syndrome: A population-based cross- Sectional study	No usable data
K. Hod; A. D. Sperber; N. Maharshak; Y. Ron; I. Shapira; Z. David; O. Rogowski; S. Berliner; S. Shenhar-Tsarfaty; R. Dekel	2018	Serum cholinesterase activity is elevated in female diarrhea-predominant irritable bowel syndrome patients compared to matched controls	No usable data
I. K. Høgestøl; M. Chahal-Kummen; I. Eribe; C. Brunborg; A. Stubhaug; S. Hewitt; J. Kristinsson; T. Mala	2017	Chronic Abdominal Pain and Symptoms 5 Years After Gastric Bypass for Morbid Obesity	No usable data
N. S. Javadekar; G. A. Oka; A. S. Joshi; P. Vaste; S. Tamane; P. S. Lawate	2021	Prevalence of irritable bowel syndrome and metabolic syndrome among young adults in an annual health check-up setting	No usable data
H. J. Jung; M. I. Park; W. Moon; S. J. Park; H. H. Kim; E. J. Noh; G. J. Lee; J. H. Kim; D. G. Kim	2011	Are Food Constituents Relevant to the Irritable Bowel Syndrome in Young Adults? - A Rome III Based Prevalence Study of the Korean Medical Students	No usable data
H. K. Jung; S. Halder; M. McNally; G. R. Locke, 3rd; C. D. Schleck; A. R. Zinsmeister; N. J. Talley	2007	Overlap of gastro-oesophageal reflux disease and irritable bowel syndrome: prevalence and risk factors in the general population	No usable data
S. S. Khayyatzadeh; A. Esmaillzadeh; P. Saneei; A. H. Keshteli; P. Adibi	2016	Dietary patterns and prevalence of irritable bowel syndrome in Iranian adults	No usable data
G. Koochakpoor; A. Salari-Moghaddam; A. H. Keshteli; A. Esmaillzadeh; P. Adibi	2021	Association of Coffee and Caffeine Intake With Irritable Bowel Syndrome in Adults	No usable data
M. Kubo; Y. Fujiwara; M. Shiba; Y. Kohata; H. Yamagami; T. Tanigawa; K. Watanabe; T. Watanabe; K. Tominaga; T. Arakawa	2011	Differences between risk factors among irritable bowel syndrome subtypes in Japanese adults	No usable data

D. A. Landau; A. Goldberg; Z. Levi; Y. Levy; Y. Niv; Y. Bar-Dayan	2008	The prevalence of gastrointestinal diseases in israeli adolescents and its association with body mass index, gender, and jewish ethnicity	No usable data
T. Larussa; L. Abenavoli; A. Corea; A. C. Procopio; L. Giubilei; R. Vallelunga; N. Polimeni; E. Suraci; R. Marasco; M. Imeneo; L. Boccuto; F. Luzza	2021	Trends and characteristics associated with dietary triggers and psychological distress in patients with irritable bowel syndrome: a cross-sectional study	No usable data
S. H. Lee; K. N. Kim; K. M. Kim; N. S. Joo	2016	Irritable bowel syndrome may be associated with elevated alanine aminotransferase and metabolic syndrome	No usable data
S. Y. Lee; H. R. Hwang; Y. H. Yi; J. M. Kim; Y. J. Kim; J. G. Lee; Y. H. Cho; Y. J. Tak; S. H. Lee; E. J. Park; Y. Lee	2021	Association between Irritable Bowel Syndrome and Risk of Osteoporosis in Korean Premenopausal Women	No usable data
S. Y. Lee; K. J. Lee; S. J. Kim; S. W. Cho	2009	Prevalence and risk factors for overlaps between gastroesophageal reflux disease, dyspepsia, and irritable bowel syndrome: A population-based study	No usable data
M. Li; B. Lu; L. Chu; H. Zhou; M. Y. Chen	2014	Prevalence and characteristics of dyspepsia among college students in Zhejiang province	No usable data
G. R. Locke Iii; A. R. Zinsmeister; S. L. Fett; L. J. Melton Iii; N. J. Talley	2005	Overlap of gastrointestinal symptom complexes in a US community	No usable data
Y. Q. Long; W. L. Xu; L. X. Li; H. Q. He; J. J. Wang; G. D. Shan; N. Dai; H. T. Chen	2024	Characteristics and Risk Factors of Functional Dyspepsia Fulfilling the Rome IV Criteria Overlapping With Gastroesophageal Reflux Disease, Irritable Bowel Syndrome, and Functional Constipation in South China	No usable data
C. L. Lu; C. Y. Chen; F. Y. Chang; S. D. Lee	1998	Characteristics of small bowel motility in patients with irritable bowel syndrome and normal humans: An Oriental study	No usable data
A. K. Mahassadi; P. C. Ebela; A. D. Bangoura; A. K. Attia	2019	The burden of irritable bowel syndrome and chronic constipation on health-related quality of life in black Africans: A comparison with healthy control subjects in Côte d'ivoire, West Africa	No usable data
M. Nagarethinam; H. Webster; S. Y. Lee; D. Con; E. Shen	2023	Functional gastrointestinal disorders among healthcare professionals at a tertiary Australian hospital	No usable data
S. Y. Nam; B. C. Kim; K. H. Ryu; B. J. Park	2010	Prevalence and risk factors of irritable bowel syndrome in healthy screenee undergoing colonoscopy and laboratory tests	No usable data
K. Otani; T. Watanabe; K. Takahashi; Y. Nadatani; S. Fukunaga; S. Hosomi; F. Tanaka; N. Kamata; K. Taira; Y. Nagami; T. Kimura; S. Fukumoto; N. Kawada; Y. Fujiwara	2021	Prevalence and risk factors of functional constipation in the Rome IV criteria during a medical check-up in Japan	Does not investigate specifically IBS

K. Otani; T. Watanabe; K. Takahashi; Y. Nadatani; M. Ominami; S. Fukunaga; S. Hosomi; N. Kamata; F. Tanaka; Y. Nagami; K. Taira; T. Kimura; S. Fukumoto; Y. Fujiwara	2023	Upper gastrointestinal endoscopic findings in functional constipation and irritable bowel syndrome diagnosed using the Rome IV criteria: a cross-sectional survey during a medical check-up in Japan	No usable data
R. Sámano; F. Esparza-Juárez; G. Chico-Barba; E. González-Medina; B. Sánchez-Jiménez; M. Hernández-Trejo	2022	Association of Diet, Body Mass Index, and Lifestyle on the Gastrointestinal Health Risk in a Sample of Adults	No usable data
H. Sharma; A. K. Verma; P. Das; S. Dattagupta; V. Ahuja; G. K. Makharia	2015	Prevalence of celiac disease in Indian patients with irritable bowel syndrome and uninvestigated dyspepsia	No usable data
R. J. Shulman; M. M. Self; D. I. Czyzewski; J. Goldberg; M. Heitkemper	2020	The Prevalence of Hypermobility in Children with Irritable Bowel Syndrome and Functional Abdominal Pain Is Similar to that in Healthy Children	No usable data
N. T. Tornkvist; I. Aziz; W. E. Whitehead; A. D. Sperber; O. S. Palsson; J. P. Hreinsson; M. Simrén; H. Törnblom	2021	Health care utilization of individuals with Rome IV irritable bowel syndrome in the general population	No usable data
P. H. Tseng; H. M. Chiu; C. H. Tu; M. S. Wu; H. N. Ho; M. J. Chen	2021	Obesity Exacerbates Irritable Bowel Syndrome-Related Sleep and Psychiatric Disorders in Women With Polycystic Ovary Syndrome	No usable data
B. Zawdie; K. H. Abate; D. Tamiru; T. Belachew	2023	Abdominal Pain of Functional Gastrointestinal Disorders in Dietary Diversity Patterns and Its Determinants among Healthy Adults in Jimma City, Southwest Ethiopia	Does not investigate specifically IBS
M. H. Zeeshan; N. P. Vakkalagadda; G. S. Sree; K. K. Anne; S. devi; O. Parkash; S. B. U. Fawwad; S. M. W. Haider; H. Mumtaz; M. Hasan	2022	Irritable bowel syndrome in adults: Prevalence and risk factors	No usable data
M. Aasbrenn; S. Lydersen; P. G. Farup	2018	A Conservative Weight Loss Intervention Relieves Bowel Symptoms in Morbidly Obese Subjects with Irritable Bowel Syndrome: A Prospective Cohort Study	No usable data
A. Alnoman; A. M. Badeghiesh; H. A. Baghlaf; M. H. Dahan	2022	Pregnancy, delivery, and neonatal outcomes among women with irritable bowel syndrome (IBS) an evaluation of over 9 million deliveries	Only investigates pregnant patients
P. Arasteh; N. Maharlouei; S. S. Eghbali; M. Amini; K. B. Lankarani; R. Malekzadeh	2018	A comprehensive look at irritable bowel syndrome and its associated factors considering the rome iv criteria: A penalized smoothly clipped absolute deviation regression approach in the pars cohort study	No usable data

P Aro, J Ronkainen, N J Talley, T Storskrubb, E Bolling-Sternevald, L Agréus	2005	Body mass index and chronic unexplained gastrointestinal symptoms: An adult endoscopic population based study	No usable data
M. Bayrak; K. Cadirci	2022	Evaluation of the Relationship Between Quality of Life, Serum 25 (OH) Vitamin D Levels, and Anxiety and Depression in Patients with Irritable Bowel Syndrome: A Case-Control Study	No usable data
K. H. Beh; K. H. Chuah; N. A. Mahamad Rappek; S. Mahadeva	2021	The association of body mass index with functional dyspepsia is independent of psychological morbidity: A cross-sectional study	No usable data
P. Grubić; D. Jurcić; B. Ebling; R. Gmajnić; B. Nikolić; S. Pribić; A. Bilić; M. T. Levak	2014	Irritable bowel syndrome in Croatia	No usable data
R. L. Levy; J. A. Linde; K. A. Feld; M. D. Crowell; R. W. Jeffery	2005	The association of gastrointestinal symptoms with weight, diet, and exercise in weight-loss program participants	No usable data
Yuanjun Dong, Sabrina Beren, Wolfgang Eich, Rainer Schaefert, Jonas Tesarz	2018	Is body mass index associated with symptom severity and health-related quality of life in irritable bowel syndrome? A cross-sectional study	No usable data
R. Sadik; E. Björnsson; M. Simrén	2010	The relationship between symptoms, body mass index, gastrointestinal transit and stool frequency in patients with irritable bowel syndrome	No usable data
S. Saneie; A. Aminianfar; F. Shidfar; A. H. Keshteli; A. Esmaillzadeh; P. Adibi	2022	The association between dietary total antioxidant capacity and odds and severity of irritable bowel syndrome among Iranian adults: a cross-sectional study	No usable data
S. Wu; Z. Yang; S. Liu; Q. Zhang; S. Zhang; S. Zhu	2024	Ultra-Processed Food Consumption and Long-Term Risk of Irritable Bowel Syndrome: A Large-Scale Prospective Cohort Study	No usable data

<u>Table S4: GRADE Evidence Profile</u>

Certainty assessment							№ of patients		Effect	Certainty
№ of studies	Study design	Risk of bias	Inconsistency	Indirectness	Imprecision	Other considerations	Overweight/Obese BMI	Normal BMI	Relative (95% CI)	
22	non-randomised studies	not serious	not serious	not serious	not serious	none	Overweight BMI 1654/60306 (2.7%)	4127/374440 (1.1%)	**OR 1.02** (0.89 to 1.17)	⨁⨁◯◯ Low
20	non-randomised studies	not serious	not serious	not serious	not serious	none	Obese BMI 848/33391 (2.5%)	4218/374422 (1.1%)	**OR 1.11** (0.91, 1.37)	⨁⨁◯◯ Low

CI: confidence interval; **OR:** odds ratio

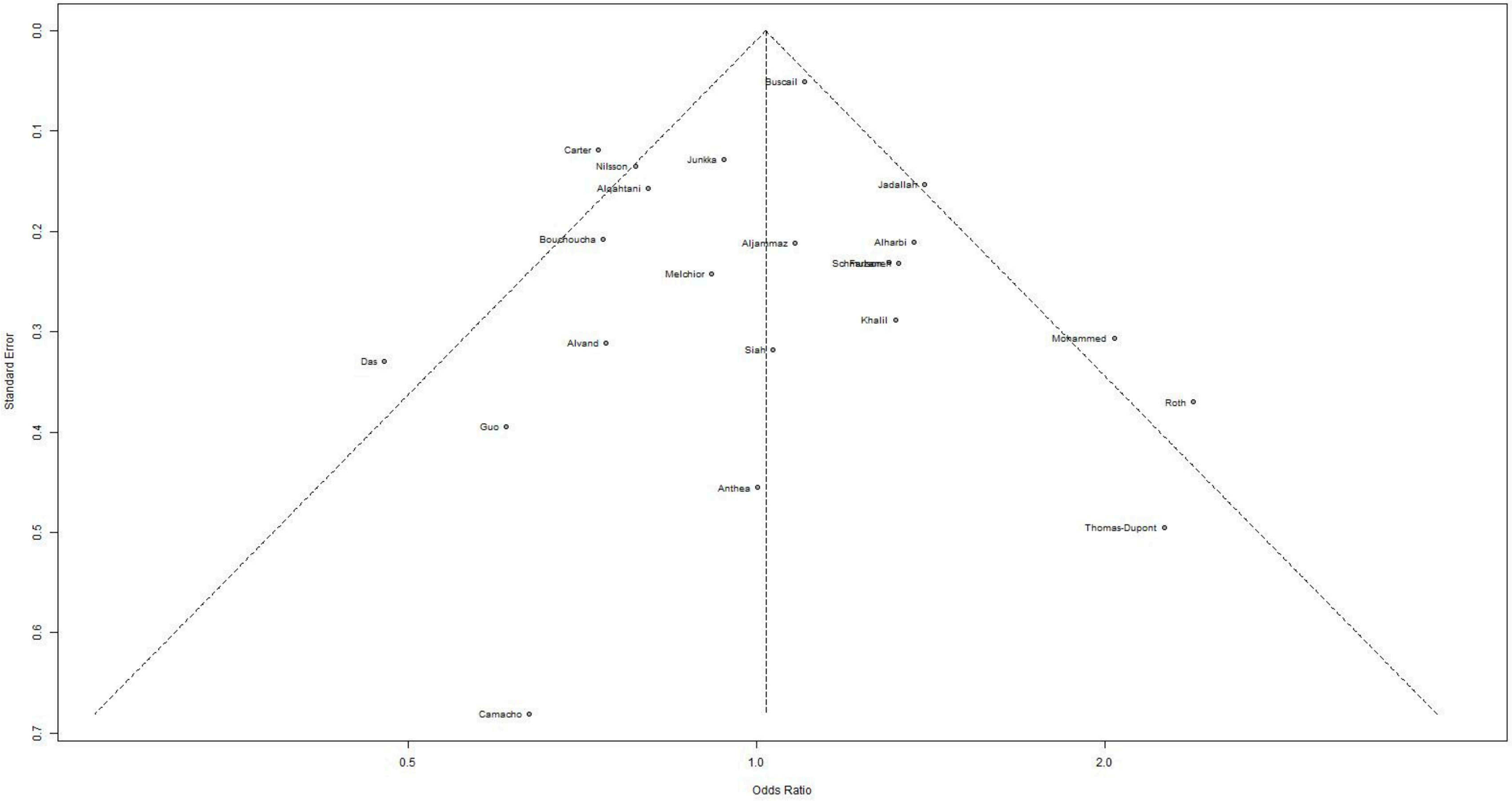

Figure S1: Funnel plot of association between overweight BMI and IBS

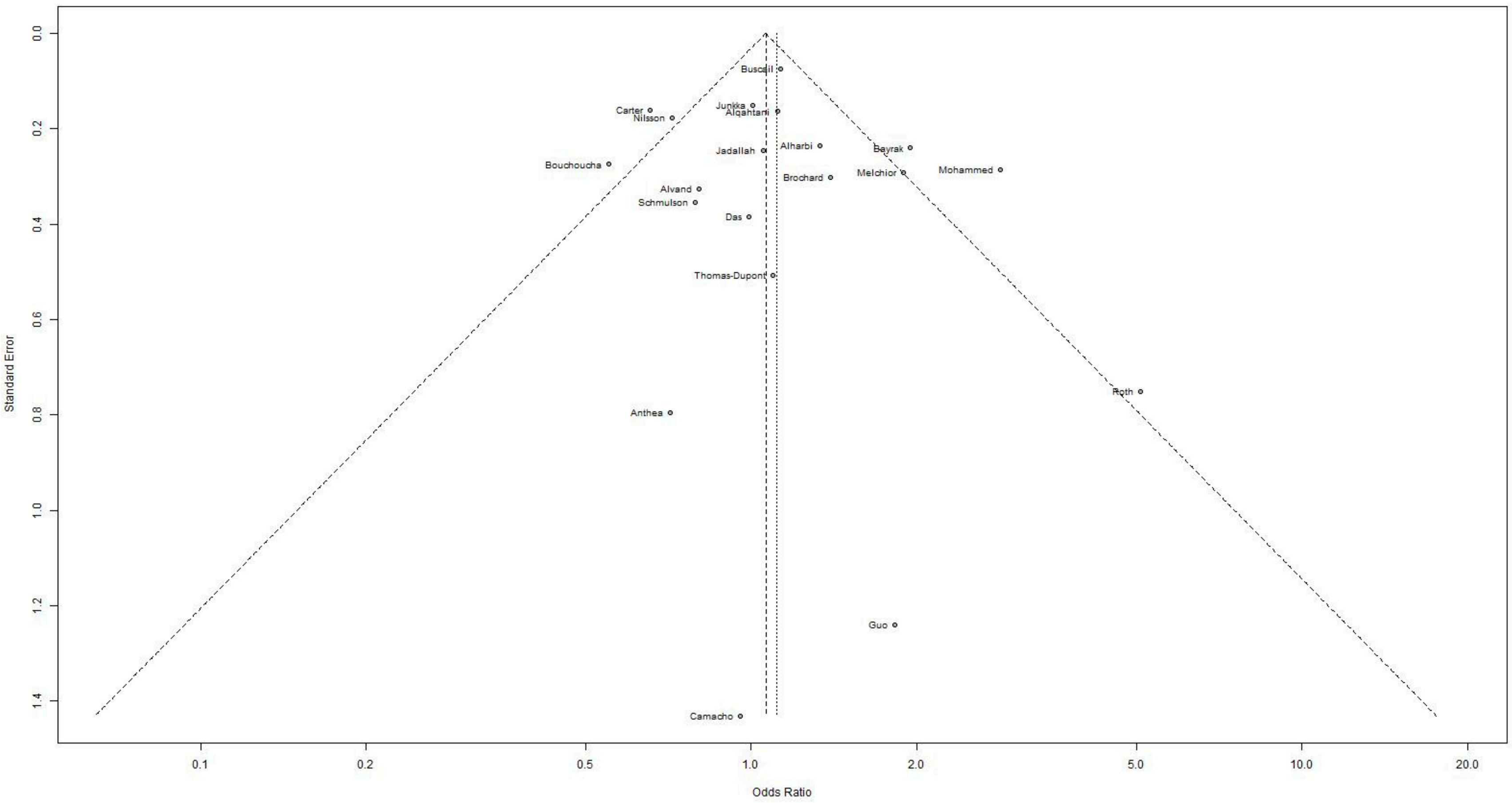

Figure S2: Funnel plot of association between obese BMI and IBS
